# Interaction of cimetidine with P450 in a mouse model of hepatocarcinogenesis initiation

**DOI:** 10.1038/sj.bjc.6600102

**Published:** 2002-02-12

**Authors:** F Caballero, E Gerez, A Batlle, E Vazquez

**Affiliations:** Centro de Investigaciones sobre Porfirinas y Porfirias (CIPYP) (CONICET - FCEN, UBA), Ciudad Universitaria, Pabellón II, 2do piso, C1428EHA Buenos Aires, Argentina

**Keywords:** hepatocarcinogenesis, cimetidine, p-dimethylaminoazobenzene, cytochrome P450, haem metabolism, oxidative stress

## Abstract

Many drugs and xenobiotics are lipophilic and they should be transformed into more polar water soluble compounds to be excreted. Cimetidine inhibits cytochrome P450. The aim of this study was to investigate the preventive and/or reversal action of cimetidine on cytochrome P450 induction and other metabolic alterations provoked by the carcinogen p-dimethylaminoazobenzene. A group of male *CF1* mice received a standard laboratory diet and another group was placed on dietary p-dimethylaminoazobenzene (0.5% w w^−1^). After 40 days of treatment, animals of both groups received p-dimethylaminoazobenzene and two weekly doses of cimetidine (120 mg kg^−1^, *i.p*.) during a following period of 35 days. Cimetidine prevented and reversed δ-aminolevulinate synthetase induction and cytochrome P450 enhancement provoked by p-dimethylaminoazobenzene. However, cimetidine did not restore haem oxygenase activity decreased by p-dimethylaminoazobenzene. Enhancement in glutathione S-transferase activity provoked by p-dimethylaminoazobenzene, persisted in those animals then treated with cimetidine. This drug did not modify either increased lipid peroxidation or diminution of the natural antioxidant defence system (inferred by catalase activity) induced by p-dimethylaminoazobenzene. In conclusion, although cimetidine treatment partially prevented and reversed cytochrome P450 induction, and alteration on haem metabolism provoked by p-dimethylaminoazobenzene AB, it did not reverse liver damage or lipid peroxidation. These results further support our hypothesis on the necessary existence of a multiple biochemical pathway disturbance for the onset of hepatocarcinogenesis initiation.

*British Journal of Cancer* (2002) **86**, 630–635. DOI: 10.1038/sj/bjc/6600102 www.bjcancer.com

© 2002 Cancer Research UK

## 

Cytochrome P450 (P450) plays a key role in the oxidative metabolism of drugs and xenobiotics, many of which are lipophilic and to be excreted they should be transformed into more polar water soluble molecules by the system of hepatic mono-oxygenases (Lim and Lu, 1998).

P450 can be influenced by a number of exogenous and endogenous factors (Whitlock and Denison, 1995) and its induction and inhibition is of the utmost interest in carcinogenesis (Toussaint *et al*, 1993).

We have earlier proposed a mechanism for the onset of hepatocarcinogenesis involving an activating status of the whole liver which reflected an important and sustained increase in P450 levels, leading to biochemical aberrations in haem metabolism which in turn would lead to the tumorigenic process (Gerez *et al*, 1997). We have also suggested that reactive oxygen species (ROS), produced during carcinogenesis chemically induced by p-dimethylaminoazobenzene (DAB), would be involved in the generation of hepatic lesions (Gerez *et al*, 1998a,b) and the so triggered peroxidative damage would be implicated in the initiation step of hepatocarcinogenesis.

The development of cancer is a dynamic process of de-regulation of gene function. Accumulation of damage alters gene function and clonal expansion of mutated cells (Cerutti, 1994). During drug metabolism, generated reactive oxygen species (ROS) play a key role in several stages of carcinogenesis (Halliwell, 1999; Caballero *et al*, 2001).

Cimetidine (CIM) is a H_2_-histamine receptor antagonist clinically used in the treatment of peptic ulcers and other gastric acid-related disorders (Chang *et al*, 1992a). It inhibits hepatic mixed-function oxidase activity (Baird *et al*, 1987) and it appears that CIM is a more potent inhibitor of hepatic P450 when administered *in vivo* than when it is added to microsomes *in vitro* (Chang *et al*, 1992b).

The presence of a high affinity binding site for CIM on P450 in liver microsomes, with both the imidazole and cyano positions of CIM interacting with the hemin iron is well documented. Ranitidine, a structurally dissimilar H_2_-histamine antagonist, not inhibiting hepatic mixed-function oxidases, has not a binding site on P450, suggesting therefore that CIM would alter the oxidative metabolism of some compounds by having a direct inhibitory effect on P450. If CIM exerts a significant general effect upon haem biosynthetic and/or degradative pathway, we would expect these effects to be manifest in both P450 and in other haem containing proteins when animals are treated with CIM (Baird *et al*, 1987).

Since elucidation of a specific form of microsomal P450 exclusively associated with azoreduction remains elusive (Zbaida, 1995), we have shown that administration of DAB to mice, remarkably increases total P450 and that these changes are associated with hepatoxicity and lipid peroxidation, leading to the carcinogenesis onset. Considering that in our experimental model, the sustained P450 induction provoked as a consequence of the carcinogen metabolism, is responsible for the liver injury and the peroxidative damage, our aim was to investigate if diminution of P450 caused by CIM could avoid the biochemical aberrations associated with the initiation stage of hepatocarcinogenesis (Gerez *et al*, 1998a,b; Caballero *et al*, 2001).

## MATERIALS AND METHODS

### Chemicals

Chemicals were reagent grade and purchased from Sigma Chemical Co. (St. Louis, MO, USA).

### Animals and treatment

**Figure 1:**
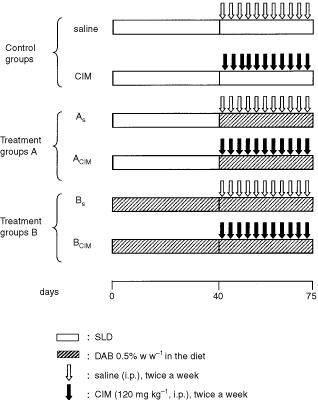
Experimental design.

Male *CF1* mice (30 g) received a standard laboratory diet (SLD, Purina 3, Asociación de Cooperativas Argentinas, San Nicolás, Buenos Aires) (groups A, *n*=16) or were placed on dietary p-dimethylaminoazobenzene (DAB, 0.5%, w/w) (groups B, *n*=16). After 40 days, animals of both groups (A and B) received DAB and two weekly doses of CIM (120 mg kg^−1^ i.p.) (A_CIM_; B_CIM_) or saline (A_S_, B_S_) for a following period of 35 days (Mera *et al*, 1994). Other group (CIM) of animals (*n*=6) received SLD along the whole period of assay and were injected with CIM under the same scheduled protocol than the DAB treated groups. Animals of control group (*n*=6) were fed with the SLD and received saline i.p. twice a week for the same period. The treatment protocol is shown in Figure 1

. All animals were given food and water *ad libitum*.

All animals were inspected at least twice daily. Body weight and food consumption were measured at intervals throughout the study. Food was removed from animals 16 h before sacrifice. Mice were killed (at least six animals per group) under ether anaesthesia at the indicated times and liver samples were processed immediately as previously described (Gerez *et al*, 1998a). All animals received human care and were treated in accordance with guidelines established by the Animal Care and Use Committee of the Argentine Association of Specialists in Laboratory Animals (AADEALC) and in accordance with the UK Guidelines for the Welfare of Animals in Experimental Neoplasia (UKCCCR, 1998).

### Assays

δ-Aminolevulinic acid synthetase (ALA-S) activity was measured as described by Marver *et al* (1966) and microsomal haem oxygenase (HO) activity according to Yoshida and Kikuchi (1978).

Cytochrome P450 (P450) content was determined in the microsomal fraction according to Omura and Sato (1964). Glutathione S transferase (GST) was determined by the method of Habig *et al* (1974). Catalase was measured as described by Chance and Maehly (1955).

The lipid peroxidation (LP) index was evaluated by the formation of malondialdehyde and determined as thiobarbituric acid reactive species (TBARS) by the method of Niehaus and Samuelson (1968). Protein concentration was determined by the method of Lowry *et al* (1951). Enzyme units (U) were defined as the amount of enzyme producing 1 nmol of product or consuming 1 nmol of substrate (catalase) under the standard incubation conditions. Specific activity (Sp. Act.) was expressed as U mg^−1^ protein.

### Statistical analysis

Newman-Keuls test was used to assess the degree of significance. A probability level of 0.05 was used in testing for significant differences between controls and treated animals.

## RESULTS

**Figure 2:**
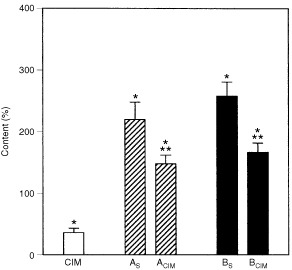
Effect of CIM on hepatic P450 levels. Animals were treated under the experimental protocol, described in Figure 1. Open bar CIM, hatched bars Treatment groups A, solid bars Treatment groups B. The data represent mean values±s.d. and are expressed as percentage of the corresponding mean control values of SLD fed animals without any other treatment, for each time point. Saline control group (mean±s.d.): P450=0.34±0.03 nmol mg^−1^ protein. Significantly different (*P*<0.05) from saline treated group * and the corresponding DAB group (A_S_ or B_S_) **.

As already shown, DAB induced a significant increase in P450 content (Gerez *et al*, 1997) and as expected CIM provoked 40% reduction in P450 levels (Nanji *et al*, 1994). Simultaneous administration of CIM and DAB (A_CIM_) partially prevented the enhancement of P450 content. When animals were pre-treated with DAB and then received CIM (B_CIM_) induction of P450 was also partially reversed. In both cases, after CIM treatment P450 levels were still 50% above basal levels ( Figure 2

).

**Figure 3:**
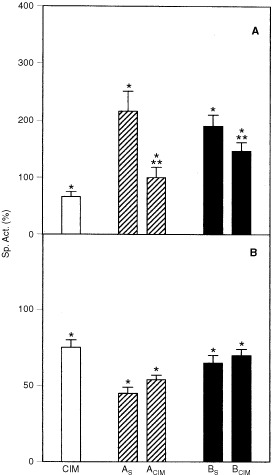
Effect of CIM on hepatic ALA-S (**A**) and HO (**B**) activities. Saline control group (mean±s.d.): ALA-S=1.4×10^−4^±0.3×10^−4^ U mg^−1^ protein; HO=2.25±0.11 U mg^−1^ protein. Other experimental conditions and symbols are as indicated in Materials and Methods and legends to Figures 1 and 2. Significantly different (*P*<0.05) from saline treated group * and the corresponding DAB group (A_S_ or B_S_) **.

It has already been reported that CIM could reduce the activity of hepatic ALA-S induced by allylisopropylacetamide (AIA) in porphyric adult rats (Marcus *et al*, 1990) and that CIM could also be used in the prophylaxis of human acute intermittent porphyria by maintaining a baseline suppression of ALA-S activity (Horie *et al*, 1995; Rogers, 1997). In this study, CIM itself inhibited 40% ALA-S activity. CIM also prevented ALA-S induction provoked by DAB restoring basal levels at the end of the treatment (A_CIM_) and even partially reversed this induction in animals pre-treated with DAB (B_CIM_), achieving a 43% diminution respect to the B_S_ group ( Figure 3a

).

CIM was found to inhibit both *in vivo* and *in vitro* the rate limiting enzymes of haem degradation, and it was suggested that the drug itself or a metabolite would inhibit HO (Marcus *et al*, 1984). In our experimental system, CIM given alone inhibited 25% HO activity and no effect was detected on the reduction of HO activity already provoked by DAB (Figure 3b).

**Figure 4:**
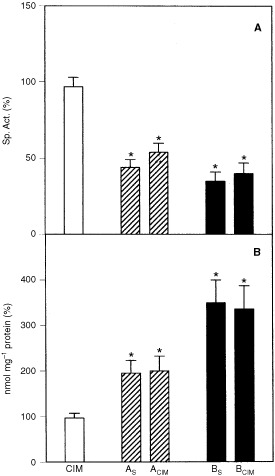
Effect of CIM on hepatic catalase activity (**A**) and LP (TBARS content, **B**). Saline control group (mean±s.d.): TBARS=135×10^−3^± 24×10^−3^ nmol mg^−1^ protein; Catalase=1.9×10^3^±0.2×10^3^ U mg^−1^ protein. Other experimental conditions and symbols are as indicated in Materials and Methods and legends to Figures 1 and 2. Significantly different (*P*<0.05) from * saline treated group and the ** corresponding DAB group (A_S_ or B_S_) .

Catalase is a haem protein present in high concentration in mammalian liver. Although administration of CIM to mice affects the synthesis and degradation of haem, no alteration in catalase activity had been described so far (Baird *et al*, 1987). In this study, CIM did not modify catalase activity in either controls or in DAB treated animals. Instead, as previously reported (Caballero *et al*, 2001) a significant inhibition has been observed in animals receiving only the carcinogen (A_S_: 50%; B_S_: 74%) ( Figure 4a

).

It was demonstrated that CIM could not prevent CCl_4_-induced LP *in vivo* (Cluet *et al*, 1986). Johnston and Kroening (1998) have observed that CIM inhibited LP in primary rats hepatocytes in culture but it did not reduce hepatocyte death induced by CCl_4_. We have found here that CIM alone did not produce any alteration in the LP index. Co-treatment (DAB+CIM) did not modify increased LP produced by DAB (A_CIM_: 110%; B_CIM_: 220%) (Figure 4b).

**Figure 5:**
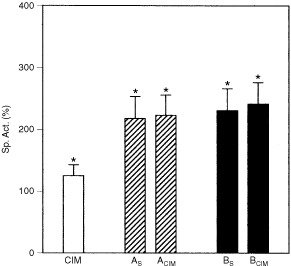
Effect of CIM on hepatic GST activity. The data represent mean values±s.d. and are expressed as percentage of the corresponding mean control values of SLD fed animals without any other treatment, for each time point. Saline control group (mean±s.d.): GST=1.45±0.25 U mg^−1^ protein. Other experimental conditions and symbols are as indicated in Materials and Methods and legends to Figures 1 and 2. Significantly different (*P*<0.05) from * saline treated group and the ** corresponding DAB group (A_S_ or B_S_).

As expected CIM produced only 25% increase in GST activity. The significant enhancement (110%) in GST activity provoked by DAB (Gerez *et al*, 1998b) persisted in those animals also receiving CIM ( Figure 5

).

## DISCUSSION

CIM a substituted imidazole, has been well documented to inhibit hepatic P450 mediated drug metabolism in rats and humans (Chang *et al*, 1992b). Numerous drug-drug interactions involving CIM have been identified, in most cases, due to inhibition of hepatic drug metabolism by CIM occurring at very low serum concentrations (Chang *et al*, 1992b). *In vitro* addition of CIM to hepatic microsomes has been shown to inhibit the P450 catalysed oxidation of many substrates, considering CIM as a general inhibitor of P450 enzymes, although Reilly *et al* (1988) have suggested that there is in fact a more selective action for CIM. It was also proposed, that the varying effects of CIM on P450 enzymes could be attributed to different CIM binding affinities for these mixed function oxidases (Faux and Combes, 1993). The inhibitory effect of CIM on the metabolic activity of CYP2C9, 2C19, 2D6 y 3A was recently demonstrated in human liver microsomes (Furuta *et al*, 2001).

P450 induction is important in the pathogenesis of alcoholic hepatic diseases and it has been demonstrated that CIM can prevent alcoholic hepatic injury by reducing LP (Nanji *et al*, 1994). Mera *et al* (1994) have also investigated whether CIM might prevent CCl_4_ induced liver cirrhosis by preventing the increase in hepatic collagen content and/or LP. They did found a protective effect of CIM, which was attributed to a reduction in P450. They have also observed that CIM stimulated the regenerative process.

Quantitative ultrastructural studies of CIM treated rat liver showed a significant proliferation of smooth endoplasmic reticulum, changes which were qualitatively similar to those produced by phenobarbital (Wright *et al*, 1991).

We have demonstrated here that CIM partially prevented and reversed the induction of P450 levels produced by the carcinogenic agent DAB. Because DAB metabolism through the mono-oxygenase system is responsible for liver damage, decrease in P450 could ameliorate or delay this process (Yan *et al*, 1998). However, the significant increase in GST activity provoked by DAB still persisted in animals also receiving CIM, indicating that reduction in P450 by CIM is not enough to completely overcome DAB toxicity and suggesting that other mechanisms would be participating in the whole process.

CIM restored ALA-S basal levels induced by DAB, but it did not reverse HO inhibition. If administration of CIM to mice would affect haem synthesis, changes in the activity of any haem protein such as catalase, would be expected. However, we did not observe any changes in either DAB treated or control animals. It has been previously demonstrated that neither acute nor chronic treatment of mice with CIM produced any effect on catalase or had any action on haem metabolism, or it did interact with any haem containing protein (Baird *et al*, 1987). Thus, both our evidence and that of others is consistent with the hypothesis proposing that CIM alters the metabolism of some compounds through its specific interaction with P450.

Therefore, as a result of DAB metabolism, ROS and reactive nitrogen species (RNS) are generated. Free radicals induced LP and the role of free radicals in carcinogenesis is also well known (Cerutti, 1994). LP index, greatly enhanced in animals fed with DAB, was not modified by CIM, consequently liver cytotoxicity should be ascribed to oxidative stress and it was here demonstrated by the persistent increase in GST activity.

We have also shown that free radicals excess was not modified by CIM in animals subjected to the carcinogenic diet, and as a consequence catalase inhibition was not affected by co-treatment of DAB and CIM. This irreversible autocatalytic process produces then a continuous increase in ROS and RNS generation leading to a desbalance between radicals and the antioxidant defence system (Pigeolet *et al*, 1990).

It is noteworthy, that increased GST, diminished catalase, and enhanced LP reflecting initiation of carcinogenesis (Gerez *et al*, 1998a,b; Caballero *et al*, 2001), were not modified by CIM, in spite of its partial reversal in the increased P450 levels.

**Figure 6:**
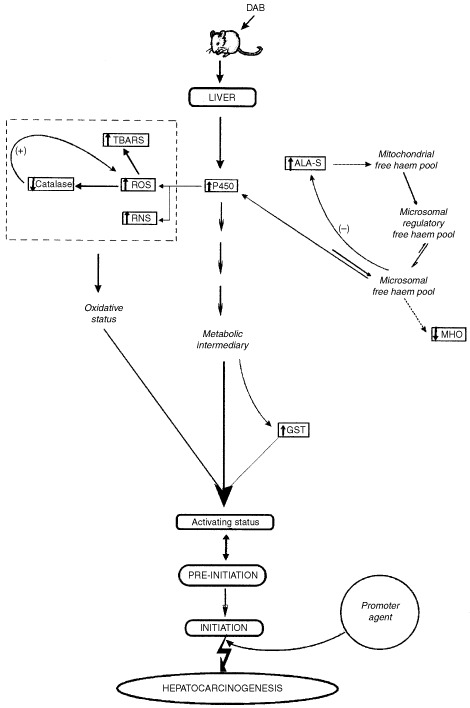
Proposed mechanism for the initiation stage of chemical induced carcinogenesis.

These results further support our hypothesis about the necessary multiple biochemical pathway disturbances for the onset of hepatocarcinogenesis initiation ( Figure 6

).

These findings also provide evidence for the importance of animal experiments in studying the multiple action and crosstalk among different biochemical pathways provoked by the administration of xenobiotics.

## References

[bib1] BairdMBSfeirGTSlade-PaciniCD1987 Lack of inhibition of mouse catalase activity by cimetidine: an argument against a relevant general effect of cimetidine upon heme metabolic pathways Biochem Pharmacol 36 43664369 3689457 10.1016/0006-2952(87)90687-3

[bib2] CaballeroFGerezEOlveriLFalcoffNBatlleAVazquezE2001 On the promoting action of tamoxifen in a model of hepatocarcinogenesis induced by p-dimethylaminoazobenzene in *CF1* mice Int J Biochem Cell Biol 33 681690 11390276 10.1016/s1357-2725(01)00056-5

[bib3] CeruttiP1994 Oxy-radicals, cancer Lancet 344 862863 7916406 10.1016/s0140-6736(94)92832-0

[bib4] ChanceBMaehlyA1955 Assay of catalases and peroxidasesInMethods Enzymology Vol 2Chance E, Maehly A (eds)pp. 764768New York: Academic Press

[bib5] ChangTLevineMBandieraSMBellwardGD1992a Selective inhibition of rat hepatic microsomal cytochrome P-450. I. Effect of the *in vivo* administration of cimetidine J Pharmacol Exp Ther 260 14411449 1545403

[bib6] ChangTLevineMBellwardGD1992b Selective inhibition of rat hepatic microsomal cytochrome P-450. II. Effect of the *in vitro* administration of cimetidine J Pharmacol Exp Ther 260 14501455 1545404

[bib7] CluetJLBoissetMBoudeneC1986 Effect of pretreatment with cimetidine or phenobarbital on lipoperoxidation in carbon tetrachloride- and trichloroethylene-dosed rats Toxicology 38 91102 3942013 10.1016/0300-483x(86)90175-7

[bib8] FauxSPCombesRD1993 Interaction of cimetidine with cytochrome P450 and effect on mixed-function oxidase activities of liver microsomes Hum Exp Toxicol 12 147152 8096713 10.1177/096032719301200209

[bib9] FurutaSKamadaESuzukiTSugimotoTKawabataYShinozakiYSanoH2001 Inhibition of drug metabolism in human liver microsomes by nizatidine, cimetidine and omeprazole Xenobiotica 31 110 11334262 10.1080/00498250110035615

[bib10] GerezEVazquezECaballeroFPoloCBatlleA1997 Altered heme pathway regulation and drug metabolizing enzyme system in a mouse model of hepatocarcinogenesis. Effect of veronal Gen Pharmac 29 569573 10.1016/s0306-3623(96)00574-5 9352304

[bib11] GerezECaballeroFVazquezEPoloCBatlleA1998a Hepatic enzymatic metabolism alterations oxidative stress during the onset of carcinogenesis: protective role of α-tocopherol Eur J Cancer Prevention 7 6976 9511853

[bib12] GerezEVazquezECaballeroFBatlleA1998b Beta-carotene partially prevents the damage induced by 1,4-dimethylaminoazobenzene Eur J Cancer Prevention 7 337342 10.1097/00008469-199808000-00010 9806123

[bib13] HabigWPabstMJakobyW1974 Glutathione S-transferases. The first enzymatic step in mercapturic acid formation J Biol Chem 249 71307139 4436300

[bib14] HalliwellB1999 Oxygen and nitrogen are pro-carcinogens. Damage to DNA by reactive oxygen, chlorine and nitrogen species: measurement, mechanism and the effects of nutrition Mutat Res 443 3752 10415430 10.1016/s1383-5742(99)00009-5

[bib15] HorieYNorimotoMTajimaFSasakiHNanbaEKawasakiH1995 Clinical usefulness of cimetidine treatment of acute relapse in intermittent porphyria Clin Chim Acta 234 171175 7758217 10.1016/0009-8981(94)05989-6

[bib16] JohnstonDEKroeningC1998 Mechanism of early carbon tetrachloride toxicity in cultured rat hepatocytes Pharmacol Toxicol 83 231239 9868740 10.1111/j.1600-0773.1998.tb01475.x

[bib17] LimJHLuAYH1998 Inhibition and induction of cytochrome P450 and the clinical implications Clin Pharmacokinet 35 361390 9839089 10.2165/00003088-199835050-00003

[bib18] LowryORosebroughNFarrARandallR1951 Protein measurement with -the Folin- phenol reagent J Biol Chem 193 265275 14907713

[bib19] MarcusDLHalbrechJLBourqueALLewGNadelHFreedmanML1984 Effect of cimetidine on delta-aminolevulinic acid synthase and microsomal heme oxygenase in rat liver Biochem Pharmacol 33 20052008 6547609 10.1016/0006-2952(84)90565-3

[bib20] MarcusDLNadelHLewGFreedmanML1990 Cimetidine suppresses chemically induced experimental hepatic porphyria Am J Med Sci 300 214217 2248274 10.1097/00000441-199010000-00003

[bib21] MarverHTschudyDPerlrothMCollinsA1966 δ-aminolevulinic acid synthetase. I. Studies in liver homogenates J Biol Chem 241 28032809 4957991

[bib22] MeraEMurielPCastilloCMourelleL1994 Cimetidine prevents and partially reverses CCl_4_-induced liver cirrhosis J Appl Toxicol 14 8790 7913103 10.1002/jat.2550140205

[bib23] NanjiAAZhaoSKhwajaSSadrzadehSMWaxmanDJ1994 Cimetidine prevents alcoholic hepatic injury in the intragastric feeding rat model J Pharmacol Exp Ther 269 832837 8182552

[bib24] NiehausWSamuelsonB1968 Formation of malonaldehyde from phospholipids arachidonate during microsomal lipid peroxidation Eur J Biochem 6 126130 4387188 10.1111/j.1432-1033.1968.tb00428.x

[bib25] OmuraTSatoR1964 The carbon monoxide binding pigment of liver microsomes J Biol Chem 239 23702378 14209971

[bib26] PigeoletECorbisierPHoubionALambertBMichielsCRaesMZacharyMRemacleJ1990 Glutathione peroxidase, superoxide dismutase and catalase inactivation by peroxides and oxygen derived free radicals Mech Ageing Dev 51 283297 2308398 10.1016/0047-6374(90)90078-t

[bib27] ReillyPEMasonSRGilliamEM1988 Differential inhibition of human liver phenacetim O-deethylation by histamine and for histamine H_2_-receptor antagonists Xenobiotica 18 381387 2899931 10.3109/00498258809041674

[bib28] RogersPD1997 Cimetidine in the treatment of acute intermittent porphyria Ann Pharmacother 31 365367 9066947 10.1177/106002809703100317

[bib29] ToussaintCAlbinNMassaadLGrunenwaldDPariseOMorizetJGouyetteAChaotG1993 Main drugs and carcinogen-metabolizing enzyme systems in human non-small cell lung cancer and peritumoral tissues Cancer Res 53 46084612 8402635

[bib30] UKCCCR-UK Coordinating Committee on Cancer Research1998 Guidelines for the Welfare of Animals in Experimental Neoplasia: London

[bib31] WhitlockJDenisonM1995 Induction of cytochrome P450 enzymes that metabolize xenobioticsInCytochrome P450: Structure, mechanisms and biochemistry2nd edition,Ortiz de Montellano P (ed)pp 367390New York: Plenum Press

[bib32] WrightAWWinzorDJReillyPE1991 Cimetidine: an inhibitor and an inducer of rat liver microsomal cytochrome P-450 Xenobiotica 21 193203 2058175 10.3109/00498259109039461

[bib33] YanYHigashiKYamamuraKFukamachiYAbeTGotohSSugiuraTHiranoTHigashiTIchibaM1998 Different responses other than the formation of DNA-adducts between the livers of carcinogen-resistant rats (DRH) and carcinogen-sensitive rats (Donryu) to 3′-methyl-4-dimethylaminoazobenzene administration Jpn J Cancer Res 89 806813 9765615 10.1111/j.1349-7006.1998.tb00632.x PMC5921908

[bib34] YoshidaTKikuchiG1978 Purification and properties of heme oxygenase from pig spleen microsomes J Biol Chem 253 42244229 96115

[bib35] ZbaidaS1995 The mechanism of microsomal azoreduction: predictions based on electronic aspects of structure-activity relationships Drug Metab Rev 27 497516 8521752 10.3109/03602539508998333

